# Evaluation and Comparison of the UV-LED Action Spectra for Photochemical Disinfection of Coliphages and Human Pathogenic Viruses

**DOI:** 10.3390/microorganisms13122798

**Published:** 2025-12-09

**Authors:** Kazuaki Mawatari, Yushi Onoda, Yasuko Kadomura-Ishikawa, Takahiro Emoto, Momoka Yamaguchi, Nozomi Hirano, Sae Toda, Mina Matsubara, Takashi Uebanso, Toshihiko Aizawa, Shigeharu Yamauchi, Yasuo Fujikawa, Tomotake Tanaka, Xing Li, Eduardo Suarez-Lopez, Richard J. Kuhn, Ernest R. Blatchley, Akira Takahashi

**Affiliations:** 1 Department of Preventive Environment and Nutrition, Institute of Biomedical Sciences, Tokushima University Graduate School, Tokushima-City 770-8503, Tokushima, Japan; yushi.onoda@nichia.co.jp (Y.O.); ishikawa-kd@tokushima-u.ac.jp (Y.K.-I.); c202431001@tokushima-u.ac.jp (M.Y.); c202431012@tokushima-u.ac.jp (N.H.); c202441002@tokushima-u.ac.jp (S.T.); matsubara@tokushima-u.ac.jp (M.M.); uebanso@tokushima-u.ac.jp (T.U.); akiratak@tokushima-u.ac.jp (A.T.); 2 Department of Microbial Control, Institute of Biomedical Sciences, Tokushima University Graduate School, Tokushima-City 770-8503, Tokushima, Japan; 3 Nichia Corporation, Anan-City 774-8601, Tokushima, Japan; toshihiko.aizawa@nichia.co.jp (T.A.); shigeharu.yamauchi@nichia.com (S.Y.); yasuo.fujikawa@nichia.co.jp (Y.F.); tomotake.tanaka@nichia.co.jp (T.T.); 4 Graduate School of Science and Technology, Tokushima University, Tokushima-City 770-8506, Tokushima, Japan; emoto@tokushima-u.ac.jp; 5 Lyles School of Civil & Construction Engineering, Purdue University, West Lafayette, IN 47907, USA; li1856@purdue.edu (X.L.);; 6 Biological Sciences, Purdue University, West Lafayette, IN 47907, USA; esuarezl@purdue.edu (E.S.-L.); kuhnr@purdue.edu (R.J.K.); 7 School of Sustainability Engineering & Environmental Engineering, Purdue University, West Lafayette, IN 47907, USA

**Keywords:** ultraviolet, UVC, Far-UVC, action spectra, coliphage, respiratory syncytial virus, human metapneumovirus, virucidal efficiency, surrogate virus

## Abstract

Ultraviolet (UV) disinfection is a powerful method for inactivating viruses. However, comparative wavelength-dependent sensitivities among human viruses and bacteriophages remain poorly characterized. Here, we evaluated the virucidal efficiencies of UV-light emitting diode (UV-LED) against multiple coliphages (MS2, Qβ, PhiX174, and T1) and mammalian viruses, including respiratory syncytial virus (RSV) and human metapneumovirus (HMPV). We used a standardized irradiation system equipped with interchangeable UV-LED modules (250–365 nm), a low-pressure mercury lamp (254 nm), and a filtered krypton-chloride excimer lamp (222 nm). All coliphages exhibited wavelength-dependent inactivation with maximal efficiency at 263–270 nm, closely matching the action spectra of RSV and HMPV (*r* > 0.94, *p* < 0.001). However, their absolute UV sensitivities were markedly lower: under 254–281 nm irradiation. RSV and HMPV were approximately 21 and 12 times more sensitive than MS2, respectively. In contrast, far-UVC (222 nm) irradiation reduced these differences, indicating simultaneous damage to viral genomes and structural proteins. These results demonstrated that coliphages and human viruses exhibit similar wavelength-dependent sensitivity to UV-LED irradiation but differ in their absolute susceptibility. Therefore, while coliphages can be conservative surrogates for evaluating UV-LED virucidal performance, their applicability to far-UVC assessments should be interpreted with caution.

## 1. Introduction

Virus disinfection methods plays a crucial role in controlling infectious disease transmission across healthcare, industrial, and public environments [1,2]. Among the available approaches, ultraviolet (UV)-based technologies are widely used because they directly damage viral nucleic acids, thereby preventing replication and subsequent infection [3,4,5]. UV radiation is categorized into UVA (315–400 nm), UVB (280–315 nm), and UVC (200–280 nm), with UVC exhibiting the strongest virucidal potency due to its efficient induction of damages in DNA and RNA [3,4,5]. Low-pressure mercury (LP-Hg) UV lamps emitting primarily at 254 nm have been the conventional choice for UV disinfection in water, air, and surface applications [4,6,7]. Recently, UV light-emitting diodes (UV-LEDs) have emerged as practical alternatives because they enable emission at discrete wavelengths without optical filtering, offer improved energy efficiency, and provide greater flexibility for precisely tuning germicidal output across diverse UV ranges [8]. To accurately compare and evaluate UV sensitivity across different viral species, we recently developed an irradiation system that allows interchangeable LEDs with peak emissions at 13 different wavelengths, ranging from 250 nm to 365 nm. The system provides standardized irradiation-related conditions, such as peak wavelength, quantifiable irradiance, narrow beam angle, bottom surface reflectivity, sample temperature, and sample volume during exposure [9]. We also developed a standardized framework for evaluating antimicrobial activity under UV-LED irradiation, enabling consistent comparison of wavelength-dependent effects across microorganisms [5]. Using this approach, we previously demonstrated that the loss of viral infectivity under UV-LED exposure is strongly correlated with viral genome damage, without any significant degradation of viral structural proteins. Across diverse mammalian viruses, wavelengths between 265 and 270 nm consistently produced the highest inactivation efficiencies, slightly longer than the traditionally expected 260-nm nucleic acid absorption peak [5]. Enveloped RNA viruses, including coronavirus, influenza A virus, respiratory syncytial virus (RSV), and human metapneumovirus (HMPV) showed greater susceptibility than non-enveloped viruses such as feline calicivirus and adenovirus [5]. Furthermore, RSV and HMPV exhibited higher susceptibility to UV-LED irradiation than other enveloped RNA viruses [5], suggesting that subtle structural or compositional differences in their envelopes or nucleocapsids might influence UV sensitivity.

Bacteriophages, also known as phages, are viruses that specifically infect and replicate within bacterial hosts. They are ubiquitous in nature and play critical roles in microbial ecology, horizontal gene transfer, and bacterial population dynamics. Importantly, bacteriophages have been used as surrogates for pathogenic viruses, particularly in studies on viral disinfection [10]. Due to their structural similarities to certain pathogenic viruses and ease of detection, they can be used to evaluate the virucidal effects of UV irradiation and other physical treatments [11,12]. Among them, coliphages, which infect coliform bacteria, are widely studied as model organisms and indicators of fecal contamination in environmental monitoring. Structurally, most bacteriophages consist of a protein capsid enclosing a nucleic acid genome—either DNA or RNA, which can be single-stranded or double-stranded—along with tail structures in many of them that facilitate host recognition and genome injection [13]. Despite the similarities between phages and eukaryotic viruses, including their status as obligate intracellular parasites that utilize host machinery for replication, they differ in their host ranges and structural complexities. Phages infect only prokaryotic cells, whereas viruses that infect humans, animals, or plants target eukaryotic cells and often possess more complex envelopes and entry mechanisms [14]. In addition, phages have simpler structures and are easier to propagate in laboratory conditions, making them ideal for evaluating methods of viral inactivation.

UV sensitivity varies markedly among bacteriophage species, reflecting differences in genome type, capsid architecture, and repair capability. For instance, single-stranded RNA (ssRNA) coliphages, such as MS2 and Qβ, are more resistant to UVC irradiation than double-stranded DNA (dsDNA) coliphages, such as T1 or T7 [15]. Weyersberg et al. demonstrated that the single-stranded DNA (ssDNA) coliphage PhiX174 showed distinct UV sensitivity compared with the double-stranded RNA (dsRNA) pseudophage Phi6 [16,17]. Furthermore, the UV sensitivity of bacteriophages is generally lower than that of pathogenic viruses, indicating that phages typically require higher fluences to achieve equivalent levels of disinfection [15,17,18]. This difference is likely attributable to their simple structural organization and robust capsid proteins, which provide greater protection to their genomes against UV-induced photochemical damage compared to the relatively fragile envelopes of many human viruses [19]. The wavelength dependence of bacteriophage inactivation has been systematically investigated to elucidate the UV action spectra for reducing viral infectivity. Beck et al. and Mamane-Gravetz et al. demonstrated that the MS2 coliphage exhibits maximal sensitivity around 260 nm when irradiated with a medium-pressure mercury lamp or a xenon lamp equipped with band-pass filters, consistent with the absorption peak of nucleic acids. Its susceptibility was markedly reduced at longer wavelengths [15,20]. This peak wavelength was slightly shorter than that reported for pathogenic viruses in our previous study, which used a standardized UV-LED irradiation system [5]. Using four different UV-LED wavelengths (254, 265, 280, and 300 nm), Rattanakul and Oguma confirmed that MS2 and PhiX174 exhibit the highest inactivation efficiency near 265 nm, with steep declines beyond 280 nm [19]. However, the differences in the virucidal efficiency of UV between bacteriophages and pathogenic viruses, as well as the associated action spectra, remain poorly understood.

Far-UVC radiation (200–230 nm), particularly the 222 nm wavelength emitted from optically filtered krypton-chloride (KrCl) excimer lamps, is considered a safe and effective alternative to conventional UV germicidal irradiation (UVGI) [21,22]. Far-UVC has been shown to efficiently inactivate a broad range of human viruses, including influenza A virus, SARS-CoV-2, and human coronaviruses, by causing photochemical damage to nucleic acids and capsid or envelope proteins [3,23,24]. The virucidal efficiency of 222 nm far-UVC irradiation is comparable to or even higher than that of conventional 254 nm UVC irradiation under equivalent fluence conditions [3,21,25]. In bacteriophages, far-UVC irradiation exhibits virucidal effects on surrogate microorganisms such as the *Pseudomonas* phage Phi6, which demonstrated approximately 2.3- to 3.9-fold greater resistance than coronaviruses [3,25]. Interestingly, Phi6 has been shown to exhibit more than 20-fold higher resistance to 254 nm UVC irradiation than coronaviruses [3,25], suggesting that bacteriophages may not always be appropriate surrogates for evaluating the virucidal effects of far-UVC irradiation. However, studies comparing the virucidal efficiencies of far-UVC and conventional UVC irradiation across bacteriophages and human pathogenic viruses are currently limited, highlighting the need for standardized comparative analyses.

This study aimed to comprehensively evaluate the wavelength-dependent virucidal effects of UV irradiation using a standardized irradiation system equipped with interchangeable UV-LED modules (250–365 nm), a low-pressure mercury (LP-Hg) lamp (254 nm), and a filtered KrCl excimer lamp (222 nm). This system enables highly controlled evaluation of spectral characteristics, irradiance uniformity, and irradiance across different UV sources under identical experimental conditions. Specifically, we aimed to (1) compare the UV sensitivities of highly susceptible human viruses, including RSV and HMPV, with those of representative coliphages used as surrogates; (2) determine the precise action spectra of UV-LED irradiation for coliphage inactivation to identify the wavelength ranges that confer maximal virucidal efficiency; and (3) compare the wavelength-dependent action spectra of UV-LED irradiation between coliphages and RSV or HMPV. Through these analyses, we aimed to elucidate the relationship between viral structure and wavelength-dependent inactivation characteristics. We also evaluated the validity of coliphages as conservative surrogates for assessing the virucidal efficacy of UV-LED and far-UVC disinfection systems.

## 2. Materials and Methods

### 2.1. Phage, Virus, and Host Cell Strains

The coliphages MS2 (NBRC 102619), T1 (NBRC 20009), Qβ (NBRC 20012), and PhiX174 (NBRC 103405), along with their host *Escherichia coli* (NBRC 13965), were obtained from the Biological Resource Center (NBRC), National Institute of Technology and Evaluation (NITE), Tokyo, Japan. The coliphages were propagated in *E. coli* cultured in LB broth (BD biosciences, Franklin Lakes, NJ, USA). The culture supernatant was then filtered through 0.22 µm polyethersulfone membrane filters and stored at −80 °C until further use. The initial viral titers of the coliphage stocks were 6.35 ± 1.23 × 10^9^ plaque forming unit (PFU)/mL (MS2), 4.78 ± 0.45 × 10^9^; PFU/mL (T1), 1.44 ± 0.43 × 10^9^ PFU/mL (Qβ), and 2.56 ± 0.25 × 10^9^; PFU/mL (PhiX174), respectively.

RSV (strain long), HMPV (strain TN/83-1211), and their host cells (Hep-2) were obtained from the American Type Culture Collection (Manassas, VA, USA), BEI Resources (Manassas, VA, USA), and the Japanese Collection of Research Bioresources (Osaka, Japan), respectively. Hep-2 cells were cultured in Dulbecco’s modified Eagle’s medium (DMEM) (Sigma-Aldrich, St. Louis, MO, USA) supplemented with 10% fetal bovine serum (Thermo Fisher Scientific, Waltham, MA, USA) and 50 µg/mL gentamicin (Fujifilm Wako Chemicals, Osaka, Japan) at 37 °C in a humidified atmosphere containing 5% CO_2_. RSV and HMPV were propagated in Hep-2 cells for 84 h at 37 °C. The culture supernatants were pre-cleared by centrifugation at 3300× *g* for 5 min, followed by filtration through 0.22 μm membrane filters. The clarified supernatants were collected and stored at −80 °C until further use. The initial viral titers of the viral stocks were 3.85 ± 1.27 × 10^6^ PFU/mL (RSV) and 1.20 ± 0.36 × 10^7^ PFU/mL (HMPV), respectively.

### 2.2. Irradiation of Phage/Virus Suspensions Using UV Lamps and LEDs

**Figure 1:**
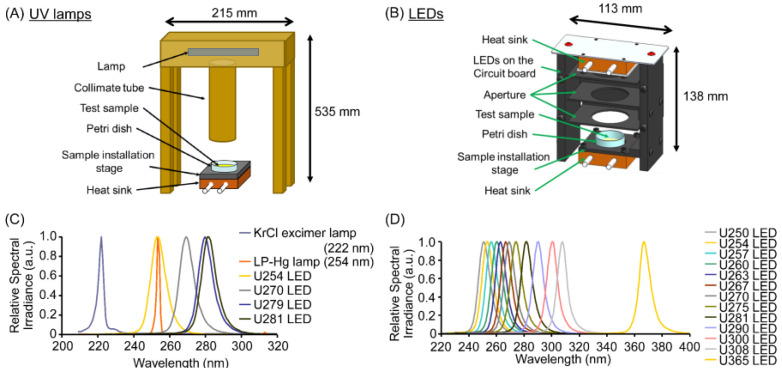
Schematic of the irradiation system equipped with a UV lamp or interchangeable LEDs at different wavelengths. (**A**) Configuration of the UV lamp system equipped with a collimator tube designed to minimize internal reflections and generate a highly collimated beam. (**B**) Configuration of the UV-LED irradiation system incorporating three low-reflective apertures and a temperature-controlled heat sink to ensure uniform and stable irradiance. (**C**) Relative spectral irradiances of the UV lamps and UV-LEDs used for Figure 2 and Figure 3 and Table 1. (**D**) Relative spectral irradiances of the UV-LEDs used for Figure 4. Detail distances from each light source to the outlet of the collimator and from the collimator outlet to the sample surface were listed in Supplementary Table S1. Peak wavelengths and irradiance values for all UV sources are summarized in Supplementary Table S2.

UV lamp irradiation was performed using an optically filtered KrCl excimer lamp (Ushio America, Cypress, CA, USA) or an LP-Hg lamp GPH212T5L/4 (ATLANTIC ULTRAVIOLET Corp., Hauppauge, NY, USA), both equipped with collimator tubes as described previously [26,27]. The collimators were specifically designed to minimize internal reflection and produce a highly collimated, near-parallel radiation beam (Figure 1A). UV-LED irradiation was performed using a standardized UV-LED irradiation system initially developed by our group [5,9,26,27]. To enhance beam collimation and suppress stray radiation, the system incorporates three apertures coated with a low-reflective material (Figure 1B). The distance between each UV source and the sample was not identical across experiments because the optical configurations differed among the light sources. Detailed distances—from each light source to the collimator outlet and from the collimator outlet to the sample surface—are provided in Supplementary Table S1. The arrangement of 36 LED chips on the circuit board was optimized through optical simulations to ensure uniform irradiation of the phage/virus suspension in a 35-mm Petri dish. To maintain a stable irradiance and peak wavelength, the LED-mounted circuit board was installed on a heat sink for temperature regulation. The irradiance was determined by measuring the photodetector output and the emission spectrum using a calibrated photodetector (S2281; Hamamatsu Photonics K.K., Shizuoka, Japan) and a spectroradiometer (C10027-01; Hamamatsu Photonics K.K.) traceable to the Japan Calibration Service System (JCSS). All measured values were confirmed to be within a ±10% error margin. Phage/virus suspensions were irradiated using UV lamps and UV-LEDs at 14 different peak wavelengths: U250, U254, U257, U260, U263, U267, U270, U275, U279, U281, U290, U300, U308, and U365 (Figure 1C,D). The spectral irradiances, actual peak wavelengths, and corresponding irradiances at the sample surface for each LED are summarized in Supplementary Table S2. After diluting each phage/virus suspension at a 1:100 ratio with phosphate-buffered saline (PBS), 1 mL of this suspension was transferred into a 35-mm Petri dish for irradiation. The sample temperature was maintained at 25 °C by connecting the heat sink beneath the sample stage to a chiller. For most LED wavelengths, the irradiance was adjusted to 1.0 mW/cm^2^ (Supplementary Table S2), resulting in a total fluence of 50 mJ/cm^2^ after 50 s of exposure. In contrast, the fluence rates of the KrCl excimer lamp and the LP-Hg lamp were 0.20 mW/cm^2^ and 0.076 mW/cm^2^, respectively (Supplementary Table S2), requiring approximately five-fold and thirteen-fold longer irradiation times to achieve the same fluence.

### 2.3. Measurements of Phage/Virus Infectivity Using Plaque-Forming Assay

The bacteriolytic activity of each phage or the cytopathic effects of each virus on host cells were evaluated using a plaque-forming unit (PFU) assay (Supplementary Figure S1), as previously reported [4,5,20]. After the phage/virus suspensions were irradiated with the UV lamp or UV-LED, the host cells were infected with 10-fold serial dilutions of the irradiated suspensions and incubated at 37 °C for 18 h (phages), 6 days (RSV), or 7 days (HMPV). A non-irradiated suspension served as the dark control. The viral inactivation efficiency of UV irradiation was assessed by calculating the log_10_ reduction in infectivity using the following formula:

log_10_(PFU/mL ratio) = log_10_(*N*_t_/*N*_0_),

where *N*ₜ is the PFU/mL of the UV-irradiated sample, and *N*_0_ is the PFU/mL of the non-irradiated control. The inactivation rate constant (*k*) was calculated using the formula:

*k* = −ln(*N*_t_/*N*_0_)/fluence (mJ/cm^2^),

based on the assumption of first-order (single-hit) inactivation kinetics.

### 2.4. Statistical Analysis

Statistical analysis was performed using analysis of variance followed by Bonferroni’s multiple comparison test with the StatView 5.0 software (SAS Institute Inc., Cary, NC, USA). Student’s *t*-test was applied to paired data where appropriate. A *p* value of <0.05 was considered statistically significant. Pearson’s correlation coefficient (*r*) and Spearman’s rank correlation test were conducted using MATLAB R2017b software (MathWorks, Natick, MA, USA) to evaluate the association between the action spectra of coliphage and virus.

## 3. Results

### 3.1. Standardized Irradiation Systems Were Validated Based on Their Virucidal Efficiency Against the MS2 Coliphage

As the UV sensitivity of the MS2 coliphage has been extensively reported, it is widely used to evaluate the virucidal effects of UV irradiation [10]. To verify whether our developed irradiation system could serve as a standardized platform for testing virucidal activity, we exposed MS2 to UV light using standard setups equipped with an optically filtered KrCl excimer lamp, an LP-Hg lamp, and U254-, U270-, U279-, and U281-LEDs. Then, we compared the resulting virucidal efficiencies with those reported previously. Table 1 summarizes the fluences of UV lamps and LEDs required to achieve 1–3 log_10_ reductions in MS2 infectivity. The virucidal efficiencies obtained using the LP-Hg lamp and UV-LEDs fell within the range of variability reported previously. These results supported the applicability of our system as a standardized method for evaluating the virucidal effects of UV-LED irradiation.

**Table 1:** Comparison of UV fluence levels required to achieve 1–3 log_10_ reductions in MS2 coliphage between previous studies and the present study.

Light Source	Peak Wavelength of LEDs (nm)	Fluence (mJ/cm^2^)			Reference
		Log_10_ Infectivity Reduction			
		−1	−2	−3	
LED	281.3	23.9	48.2	-	This study
	280	24	46	73	Oguma et al. [28]
	279.1	23.0	46.3	-	This study
	275	25	55	-	Bowker et al. [29]
	269.3	16.1	33.5	-	This study
	265	22	59	93	Oguma et al. [28]
	265	16	35	-	Sholtes et al. [30]
	265	16	33	52	Song et al. [31]
	255	19	43	72	Simons et al. [32]
	255	25	50	-	Bowker et al. [29]
	253.3	19.7	41.1	62.6	This study
LP-Hg lamp	254	29.4	-	-	Weng et al. [33]
	254	31.1	-	-	Zyara et al. [34]
	253.44	28.4	-	-	This study
KrCl excimer lamp	222	6.5	13.6	21,8	Hull et al. [35]
	222	7.4	14.7	22.1	Beck et al. [6]
	222.03	11.1	21.6	32.1	This study

LED, light-emitting diode; LP-Hg, low-pressure mercury; KrCl, krypton–chloride.

In contrast, the fluence required for infectivity reduction by 222 nm far-UVC irradiation using an optically filtered KrCl excimer lamp was approximately 1.5 times higher in this study than that reported previously by Beck et al. [6] and Hull et al. [35]. For example, they reported that a 7.4 mJ/cm^2^ fluence was required for a 1.0 log_10_ reduction compared to 11.1 mJ/cm^2^ in our study. Although both the optical thickness and the composition of the virus suspension can influence virucidal sensitivity, we confirmed that all viral suspensions used in this study exhibited high transmittance across the far-UVC to visible-light range (Supplementary Figure S2). Furthermore, all virus suspensions in our experiments were diluted more than 100-fold in PBS, consistent with the protocols used by Beck et al. and Hull et al. The suspension thickness in our experiments was approximately five-fold lower than that used by Beck et al., indicating that differences in suspension thickness or composition are unlikely to account for the higher fluence required in our study. Therefore, these factors appear independent of the observed discrepancy in far-UVC sensitivity between our results and previous reports. Beck et al. and Hull et al. measured irradiance using a commercially available radiometer (ILT-1700; International Light Technologies, Peabody, MA, USA). In our study, the irradiance was determined by measuring the photodetector output and the emission spectrum using a calibrated photodetector and as a spectroradiometer, which is traceable to the JCSS. The total spectral output was corrected according to the spectral responsivity of each wavelength (Figure 1C), yielding an average irradiance of 0.076 mW/cm^2^ at a distance of 70 mm from the outlet of the collimating tube (Figure 1A). Conventional radiometers are typically calibrated for 100% spectral response at a single reference wavelength. Therefore, when measuring wavelengths differing from the calibration reference, the indicated irradiance might be lower than the true irradiance, depending on the detector’s spectral responsivity. The irradiation can be estimated more accurately by combining direct photodetector measurements with spectral confirmation from the multichannel analyzer. Additionally, unlike the continuous emission of LEDs, the filtered KrCl excimer lamp emits pulsed radiation at a period of approximately 10 μs, as confirmed by oscilloscope monitoring (Supplementary Figure S3A). Spectral analysis in the 200–1000 nm range revealed a dominant emission peak at 222 nm with a minor component between 700 and 900 nm (Supplementary Figure S3B). The emission above 700 nm did not exert any antimicrobial activity (Supplementary Figure S3C), which was consistent with a previous report [36]. We therefore calculated the effective irradiance by excluding the photodetector output corresponding to this wavelength. These methodological differences likely account for the discrepancies in irradiance values between our study and previous reports.

### 3.2. UV Sensitivities of the MS2 Coliphage and Viruses Differed Under Standardized Irradiation

**Figure 2:**
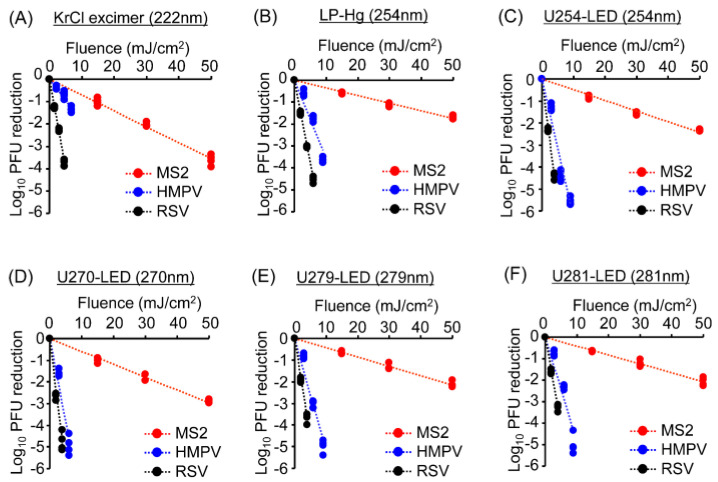
Reductions in phage and virus infectivity after irradiation with different wavelengths of UV lamps and LEDs. (**A**–**F**) Infectivity reduction in MS2 phage, RSV, and HMPV following irradiation with a (**A**) filtered KrCl excimer lamp, (**B**) LP-Hg lamp, (**C**) U254-LED, (**D**) U270-LED, (**E**) U279-LED, or (**F**) U281-LED. Host cells were infected with phage or virus suspensions that were irradiated at the indicated fluences. Infectivity was assessed by PFUs, and infectivity reduction is shown as the log_10_ PFU ratio (n = 4–6). RSV, Respiratory syncytial virus; HMPV, Human metapneumovirus; LED, light-emitting diode; UV, ultraviolet; LP-Hg, low-pressure mercury; PFU, plaque-forming unit.

To compare UV sensitivity across various wavelengths between phages and viruses, we irradiated MS2 coliphage, RSV, and HMPV using an optically filtered KrCl excimer lamp, an LP-Hg lamp, and UV-LEDs with peak emissions at 254, 270, 279, and 281 nm under standardized antimicrobial irradiation setups. MS2 suspensions were exposed to fluences of 15, 30, and 50 mJ/cm^2^, whereas RSV and HMPV suspensions were irradiated at fluences of 2, 4, and 6 mJ/cm^2^. The infectivity of phage and viruses was reduced in a fluence-dependent manner, showing approximately 2–5 log_10_ reductions A–E and Figure S1). RSV consistently showed higher UV sensitivity than HMPV across all irradiation sources (Figure 2), consistent with our previous report using LED irradiation [5]. In contrast, MS2 coliphage exhibited markedly greater resistance to UV radiation compared to the viruses.

**Figure 3:**
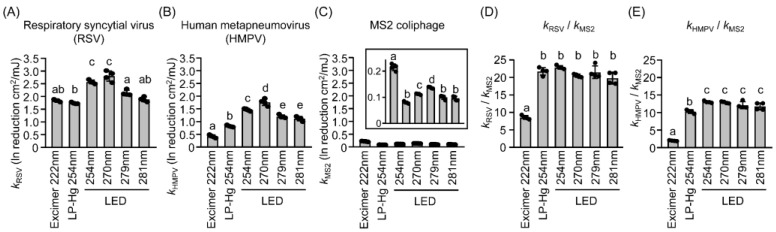
Inactivation constants (*k*) calculated after irradiation with different wavelengths by UV lamps and LEDs for (**A**) respiratory syncytial virus, (**B**) human metapneumovirus, and (**C**) the MS2 coliphage. *k* ratios of (**D**) RSV to MS2 coliphage and (**E**) HMPV to MS2 coliphage. The *k* values represent the reduction in infectivity per unit fluence for viruses or MS2 coliphage. *k* was calculated as the natural logarithm of the reduction in PFUs divided by the applied fluence. The box corresponds to the magnified graph shown in the inset. Data are presented as mean ± SD (n = 4–6). Different letters (a–e) indicate statistically significant differences (*p* < 0.05) based on ANOVA followed by Bonferroni’s multiple comparison test. LED, light-emitting diode; PFU, plaque-forming unit; ANOVA, analysis of variance.

To further compare the virucidal efficiency of different UV irradiations, we calculated inactivation constants (*k*) for RSV (*k*_RSV_), HMPV (*k*_HMPV_), and MS2 (*k*_MS2_) (Figure 3). Among the tested wavelengths, 270 nm LED irradiation produced the highest *k* values for RSV and HMPV (Figure 3A,B, respectively), indicating superior virucidal efficiency at this wavelength. In contrast, the 222 nm KrCl excimer lamp yielded the highest *k* value for MS2 (Figure 3C). When comparing the k ratios of viruses to coliphages (*k*_RSV_/*k*_MS2_ and *k*_HMPV_/*k*_MS2_), MS2 was approximately 21-fold and 12-fold more resistant than RSV and HMPV, respectively, under LP-Hg and LED irradiation. However, for the 222 nm KrCl excimer lamp, the *k*_RSV_/*k*_MS2_ and *k*_HMPV_/*k*_MS2_ ratios were significantly lower than for the other UV sources. These results demonstrated that MS2 coliphage is more resistant to UV irradiation than RSV and HMPV. However, its sensitivity profile differs from those of mammalian viruses specifically for 222 nm irradiation.

### 3.3. UV Sensitivities of Coliphages and Viruses Differed Across Wavelengths

**Figure 4:**
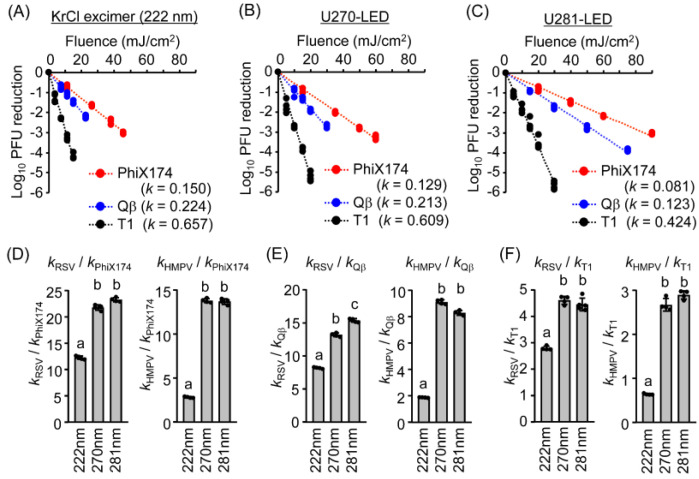
Reduction in viral infectivity against coliphages after irradiation with different wavelengths of UV lamps and light-emitting diodes (LEDs). (**A**–**C**) Infectivity reduction in PhiX174, Qβ, and T1 phages following irradiation with (**A**) 222 nm KrCl excimer lamp, (**B**) 270 nm LED, or (**C**) 281 nm LED. (**D**–**F**) Ratios of inactivation rate constants (*k*), representing infectivity reduction per unit fluence, for pathogenic virus (RSV or HMPV) relative to coliphages. *k* ratios (**D**) of pathogenic virus to PhiX174 for KrCl excimer lamp or LED irradiation; (**E**) pathogenic virus to Qβ; (**F**) pathogenic virus to T1. Data are presented as mean ± SD (n = 4–6). Different letters (a–c) indicate statistically significant differences (*p* < 0.05) based on ANOVA followed by Bonferroni’s multiple comparison test. LED, light-emitting diode; RSV, respiratory syncytial virus; HMPV, human metapneumovirus; KrCl, krypton–chloride; SD, standard deviation; ANOVA, analysis of variance.

To further confirm the differences in virucidal efficiency between coliphages and mammalian viruses, we irradiated additional coliphages—PhiX174, Qβ, and T1—using a KrCl excimer lamp and UV-LEDs with peak emissions at 270 and 281 nm under standardized antimicrobial irradiation setups. The infectivity of these phages was reduced in a fluence-dependent manner, showing approximately 3–6 log_10_ reductions (Figure 4A–C). The UV resistance exhibited by Qβ was similar to that of MS2 across the KrCl excimer lamp and UV-LEDs (Figure 3 and Figure 4). Among the coliphages, PhiX174 exhibited the highest resistance, whereas T1 showed the lowest resistance to UV irradiation (Figure 4).

Next, we calculated the inactivation constants (*k*) for PhiX174 (*k*_PhiX174_), Qβ (*k*_Qβ_), and T1 (*k*_T1_), and compared the ratios of viral to coliphage constants (Figure 4A–F). Under 270 nm and 281 nm LED irradiation, PhiX174 was approximately 22-fold and 14-fold more resistant than RSV and HMPV, respectively (Figure 4D). In contrast, the *k*_RSV_/*k*_PhiX174_ and *k*_HMPV_/*k*_PhiX174_ ratios were significantly lower for the 222 nm KrCl excimer lamp than for LED irradiation (Figure 4D), consistent with the trend observed for MS2 (Figure 3B). Similarly, Qβ and T1 exhibited lower viral-to-coliphage efficiency ratios under 222 nm irradiation compared to the other wavelengths (Figure 4E,F). These results showed that coliphages are more resistant to UV irradiation than mammalian viruses. However, their relative sensitivities varied by phage species, with PhiX174 being the most resistant and T1 the least resistant, and by irradiation wavelength, with coliphages showing comparatively higher sensitivity to 222 nm irradiation than mammalian viruses but not differing significantly under 254–281 nm irradiation. A similar discrepancy was previously reported for the *Pseudomonas* phage Phi6 [3,25]. Taken together, these findings suggested that bacteriophages cannot serve as complete surrogates for mammalian viruses for evaluating the virucidal effects of 222 nm or far-UVC irradiation.

### 3.4. The UV Action Spectra for Virucidal Efficiency Were Similar for Coliphages and Mammalian Viruses

**Figure 5:**
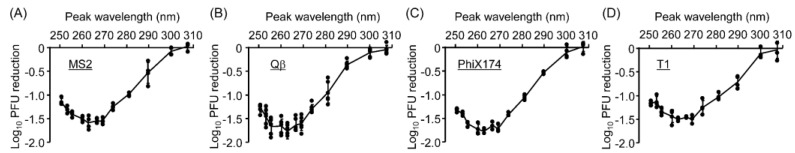
UV action spectra of LEDs for infectivity reduction for different coliphages, such as (**A**) MS2, (**B**) Qb, (**C**) PhiX174, and (**D**) T1. The coliphage suspensions were irradiated with LEDs at each peak wavelength using fluences of (**A**) 25 mJ/cm^2^, (**B**) 18 mJ/cm^2^, (**C**) 27 mJ/cm^2^, and (**D**) 5 mJ/cm^2^, corresponding to the fluences required to achieve approximately a 1.0 log_10_ reduction in infectivity under U281-LED irradiation. Coliphage infectivity was measured using PFU assays. Infectivity reduction is presented as the log_10_ ratio of PFUs. Values are shown as mean ± SD (n = 4). LED, light-emitting diode; PFU, plaque-forming unit.

We previously established the UV action spectra for reducing the infectivity of pathogenic viruses, including coronaviruses, influenza A virus, RSV, HMPV, herpes simplex virus, and feline calicivirus, using a standardized system equipped with interchangeable LEDs at 13 different peak wavelengths (250–365 nm). We also determined the peak virucidal efficiencies at 263–270 nm for all tested viruses [5]. To determine whether the UV action spectra of coliphages resemble those of mammalian viruses, we irradiated four coliphages (MS2, PhiX174, Qβ, and T1) using the same system and compared their action spectra with those of RSV and HMPV. Phage suspensions were irradiated at fluences adjusted for their relative UV resistance: 25, 27, 18, and 5 mJ/cm^2^ for MS2, PhiX174, Qβ, and T1, respectively (Figure 5). All coliphage strains exhibited similar action spectra, with measurable reductions in infectivity following irradiation at wavelengths of ≤290 nm. Among these, LEDs with wavelengths of 263–270 nm consistently showed the highest virucidal efficiency for all coliphages. However, this efficiency declined progressively from 260 nm to 250 nm, with shorter wavelengths being relatively less effective for inactivation. These findings suggested that the virucidal effects are strongly wavelength-dependent and that wavelengths below 260 nm might be less effective for inactivating coliphages.

To further evaluate the similarity between coliphages and mammalian viruses, we calculated the Pearson’s correlation coefficients (*r*) between the action spectra for infectivity reduction in the four coliphages (MS2, PhiX174, Qβ, and T1) and those of RSV and HMPV. The *r* values exceeded 0.94 (*p* < 0.001) for all comparisons (Table 2), indicating that the wavelength-dependent inactivation profiles of coliphages and mammalian viruses are highly similar, despite differences in absolute UV resistance.

## 4. Discussion

This study systematically evaluated the UV sensitivity of coliphages and human pathogenic viruses under standardized irradiation conditions using UV-LEDs, LP-Hg lamps, and far-UVC (222 nm) KrCl excimer lamps. The results revealed distinct differences in the absolute virucidal efficiency and wavelength dependency among the tested phages, as well as between phages and mammalian viruses. Furthermore, our findings confirmed that UV susceptibility varies significantly among coliphage species, which can be attributed to the differences in genome type, capsid architecture, and protein composition.

The UV susceptibility of the four test coliphages was in the following order: T1 > MS2 ≈ Qβ > PhiX174, with T1 being the most sensitive and PhiX174 the most resistant (Figure 3 and Figure 4). While these results are broadly consistent with previous observations that structural and genomic characteristics influence UV resistance, it should be noted that viral sensitivity to UVC radiation is dependent on multiple factors. In particular, the size and composition of the viral genome (DNA or RNA) are critical for determining UV susceptibility. Previous studies quantitatively described this relationship using a genomic model that predicted viral inactivation based on nucleic acid composition [17,21,37,38]. When compared with enveloped mammalian ssRNA viruses, all non-enveloped coliphages exhibited markedly lower UV sensitivity. Under conventional UVC lamp and LED irradiation (254–281 nm), the UV sensitivity of RSV and HMPV was approximately 21-fold and 12-fold higher, respectively, than that of MS2 (Figure 3). Similar observations were made when comparing the present data with previous reports for other enveloped ssRNA viruses, including coronaviruses and influenza A viruses, which showed substantially higher susceptibility to UV irradiation than any of the non-enveloped coliphages (Supplementary Figure S4). Taken together, these findings demonstrated that enveloped RNA viruses are more susceptible to UVC exposure than non-enveloped phages because of their fragile lipid envelopes and surface glycoproteins that are readily degraded by photochemical reactions [3,5,19,39]. In contrast, the greater UV resistance of coliphages can be attributed to their dense capsid proteins, which have a low content of aromatic amino acids, effectively shielding the enclosed genome from UVC irradiation and mitigating UV-induced nucleic acid photodamage [40].

Interestingly, under far-UVC irradiation at 222 nm, the differences in the UV sensitivities between phages and viruses were substantially lower. The inactivation rate constants (*k*) ratios, *k*_RSV_/*k*_MS2_ and *k*_HMPV_/*k*_MS2_, decreased by 4.7- and 3.2-fold, respectively, indicating that 222 nm far-UVC irradiation inactivated phages more efficiently than viruses. Similar findings were reported by Beck et al., who demonstrated that 222 nm irradiation simultaneously induced nucleic-acid and protein damage in human adenovirus [41,42]. The enhanced UV resistance of viruses can be attributed to their robust capsid structures, which are rich in β-sheets and have a relatively low density of aromatic amino acids [21,43]. Aromatic residues such as tryptophan and tyrosine are known to absorb UV photons, leading to protein degradation and loss of infectivity [44]. Consequently, far-UVC affects the genome and structural components, whereas UVC wavelength primarily targets nucleic acids.

The action spectra obtained from 13 different LED wavelengths demonstrated that all coliphages exhibited maximal virucidal efficiency between 263 and 270 nm, followed by a steep decline at wavelengths above 280 nm. Pearson’s correlation coefficients (*r*) between the LED action spectra of the coliphages and those of RSV or HMPV all exceeded 0.94 (*p* < 0.001, Table 2), confirming a strong similarity in wavelength-dependent response patterns. Previous studies reported similar correlations between the action spectra of bacteriophages and eukaryotic viruses [5,19]. This finding suggested that, although phages differ in overall sensitivity, their relative wavelength-response curves closely resemble those of mammalian viruses under LED irradiation.

In summary, these results indicated that coliphages and human viruses share similar wavelength-dependent action spectra under UV-LED irradiation but differ markedly in absolute UV susceptibility. Nevertheless, as coliphages exhibit lower UV sensitivity, lack of pathogenicity to humans, and reproducible photoinactivation behavior, they are widely recognized as conservative and safe surrogate models for evaluating the virucidal efficacy of UV sources under standardized irradiation conditions. Their predictable wavelength-dependent responses enable reliable benchmarking of UV-LED performance and facilitate cross-study comparisons of disinfection efficiency.

Recently, far-UVC irradiation has gained popularity due to its unique optical properties and promising potential for UVGI in occupied environments. In addition to the 222 nm filtered KrCl excimer lamp, new light sources, such as far-UVC LEDs and Luminous Array Film (LAFi) devices, which excite phosphors via plasma discharge in neon-xenon gas mixtures within sealed glass tubes, have been developed to enhance emission efficiency and scalability [45,46]. However, under far-UVC irradiation, the relative resistance of coliphages decreases, and their inactivation behavior no longer represents a conservative boundary for human viruses. Therefore, while coliphages can serve as conservative surrogates for evaluating the virucidal effects of UV-LED, their suitability for modeling far-UVC disinfection remains limited and requires further validation.

## 5. Conclusions

This study highlights the wavelength-dependent differences in UV susceptibility between coliphages and human pathogenic viruses. While the UV action spectra of coliphages are similar to those of mammalian viruses under LED irradiation, their structural resilience leads to higher resistance, especially at conventional UVC wavelengths. However, far-UVC (222 nm) irradiation can simultaneously damage viral genomes and structural proteins, thereby diminishing the protective advantage of the robust phage capsid. Our findings emphasized the importance of selecting suitable surrogate organisms based on UV sources and target wavelengths. T1 coliphage might be a suitable conservative surrogate for LED-based disinfection systems, but its applicability to far-UVC systems should be carefully validated. These findings provide essential wavelength-specific information that can support the optimization of UV-LED and far-UVC disinfection systems. By identifying conservative surrogate phages and their spectral profiles, our results may guide the development of more efficient and evidence-based strategies for controlling viral pathogens in healthcare, water treatment, and air-disinfection applications. Future studies should explore the molecular mechanisms underlying far-UVC-induced protein damage and expand comparative analyses across a broader range of viral and phage species.

## Figures and Tables

**Table 2:** Pearson’s correlation coefficients (*r*) between UV-LED action spectra of pathogenic viruses and coliphages.

Coliphage Strain	Pathogenic Virus [5]			
	RSV		HMPV	
	*r*	*p*	*r*	*p*
MS2	0.9888	<0.001	0.9681	<0.001
Qb	0.9978	<0.001	0.9739	<0.001
PhiX174	0.9918	<0.001	0.9705	<0.001
T1	0.9666	<0.001	0.9454	<0.001

The UV-LED action spectra data for pathogenic viruses were referenced from a previous study [1]. RSV, respiratory syncytial virus; HMPV, human metapneumovirus.

## Data Availability

The original contributions presented in this study are included in the article/Supplementary Material. Further inquiries can be directed to the corresponding author (K.M.).

## Supplementary Materials

The following supporting information can be downloaded at: https://www.mdpi.com/article/10.3390/microorganisms13122798/s1, Table S1: Distances from each light source to the outlet of the collimator and from the collimator outlet to the sample surface. Table S2: Peak wavelength and irradiance of UV lamps and light-emitting diodes (LEDs) used in this study; Figure S1: Related to Figure 2. Reduction in virus infectivity induced by UV irradiation; Figure S2: Transmittance of viral suspensions across the far-UVC to visible-light range; Figure S3: Pulse emission and spectral irradiance of the filtered KrCl excimer lamp (222 nm); Figure S4: Summary of viral genome or envelope type and inactivation rate constants (*k*) of mammalian viruses and coliphages irradiated with 281 nm light-emitting diodes (LEDs).

## Abbreviations

The following abbreviations are used in this manuscript:

DMEM	Dulbecco’s modified Eagle’s medium
dsDNA	Double-stranded DNA
dsRNA	Double-stranded RNA
HMPV	Human metapneumovirus
JCSS	Japan Calibration Service System
KrCl	Krypton–chloride
LED	Light-emitting diode
LP-Hg	Low-pressure mercury
NBRC	Biological Resource Center
NITE	National Institute of Technology and Evaluation
PBS	Phosphate-buffered saline
PFU	Plaque-forming unit
RSV	Respiratory syncytial virus
ssDNA	Single-stranded DNA
ssRNA	Single-stranded RNA
UV	Ultraviolet
UV-LED	UV-light emitting diode
UVGI	UV germicidal irradiation
