# Supplementary material for: Evaluation and Comparison of the UV-LED Action Spectra for Photochemical Disinfection of Coliphages and Human Pathogenic Viruses
Source: Microorganisms. 2025 Dec 9;13(12):2798. doi: 10.3390/microorganisms13122798 (PMC12736174; doi:10.3390/microorganisms13122798)
Supplement: Supplementary file 1 [file microorganisms-13-02798-s001.zip › microorganisms-4005570-supplementary.pdf]

Supplementary Table S1. Distances from each light source to the outlet of the collimator and from the collimator outlet to the sample surface.

Light source	Distance (mm)		
	From light source to collimator outlet	From collimator outlet to sample surface	Total distance
KrCl excimer lamp	380	70	450
Hg-UV lamp	325	125	450
LEDs	69	31	100

Supplementary Table S2. Peak wavelength and fluence rate of UV lamps and light-emitting diodes (LEDs) used in this study.

UV lamp / LED (spec)	Peak wavelength (nm) – measured	Fluence rate (mW/cm^2)
KrCl excimer lamp	222.03	0.076
Hg-UV lamp	253.44	0.2
U250-LED	250.8	0.5
U254-LED	253.3	1.0 (0.2*)
U257-LED	255,7	1.0
U260-LED	260.3	1.0
U263-LED	263.0	1.0
U267-LED	266.8	1.0
U270-LED	269.3	1.0
U275-LED	274.0	1.0
U279-LED	279.1	1.0
U281-LED	281.3	1.0
U290-LED	289.7	1.5
U300-LED	300.2	1.0
U308-LED	307.5	1.0
U365-LED	367.0	18.0

*The fluence rate for the experiments shown in Table 1 and Figures 2–3 was 0.2 mW/cm^2

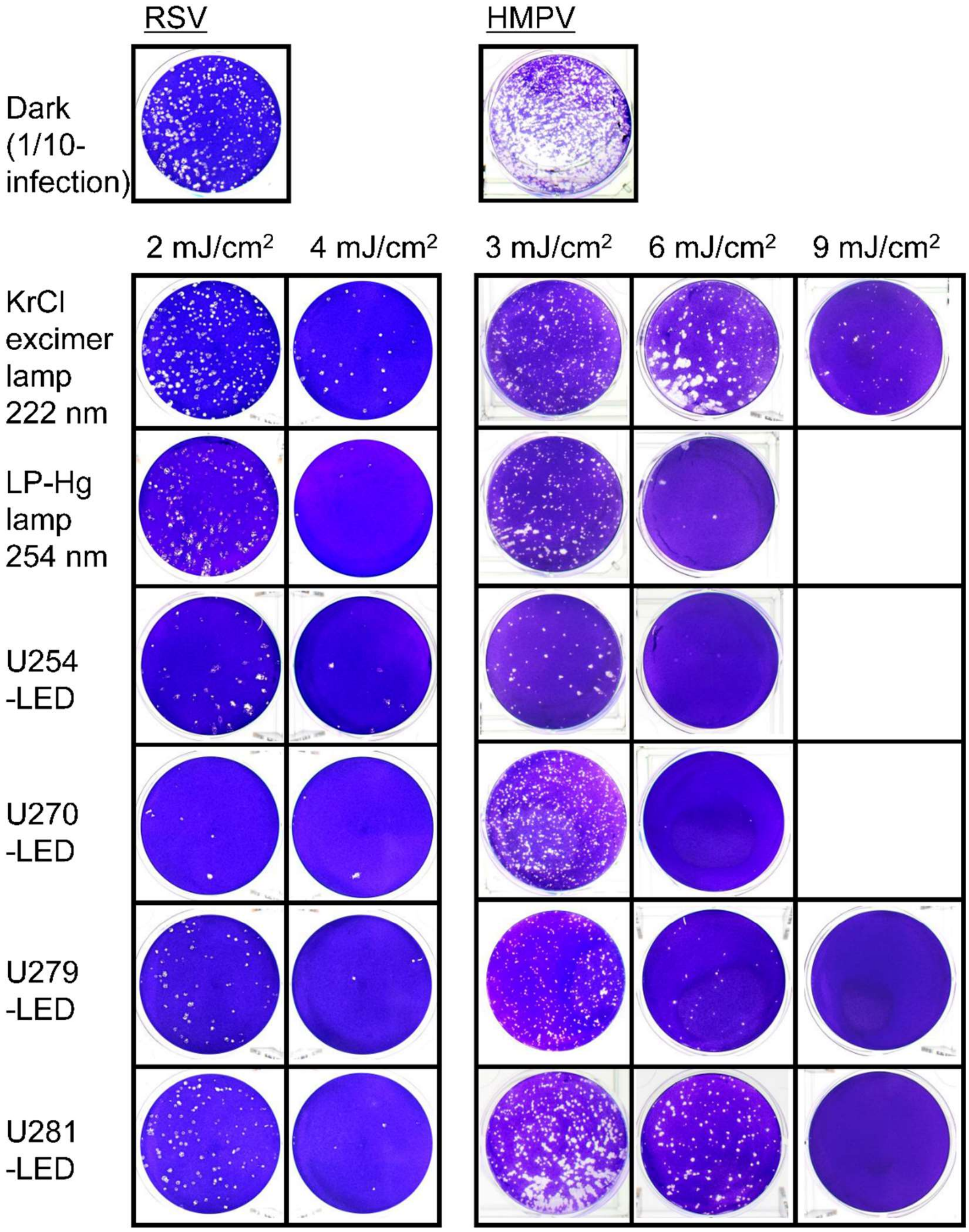

Supplementary Figure S1. Related to Figure 2. Reduction in virus infectivity induced by UV irradiation. Representative images of plaque-forming assays used to evaluate the virucidal effects of UV-lamp and UV-LED irradiation. Shown are results for respiratory syncytial virus (RSV, left panels) and human metapneumovirus (HMPV, right panels). Virus suspensions were irradiated with either a UV lamp or UV-LED at the indicated fluences and subsequently used to infect host cells. Viral infectivity was determined by quantifying plaque-forming units (PFU).

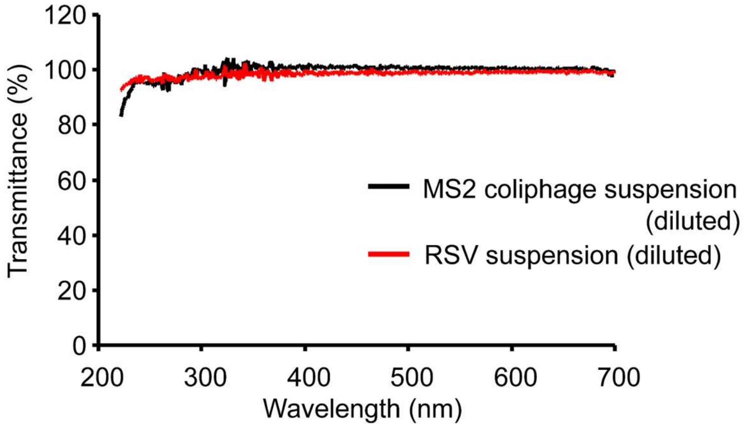

Supplementary Figure S2. Transmittance of viral suspensions across the far-UVC to visible-light range. The transmittance (%) of diluted viral suspensions is shown relative to Milli-Q water. Transmittance measurements were performed using a spectrophotometer (DU730; Beckman Coulter, Brea, CA, USA) equipped with a 1-mm optical-path-length cuvette.

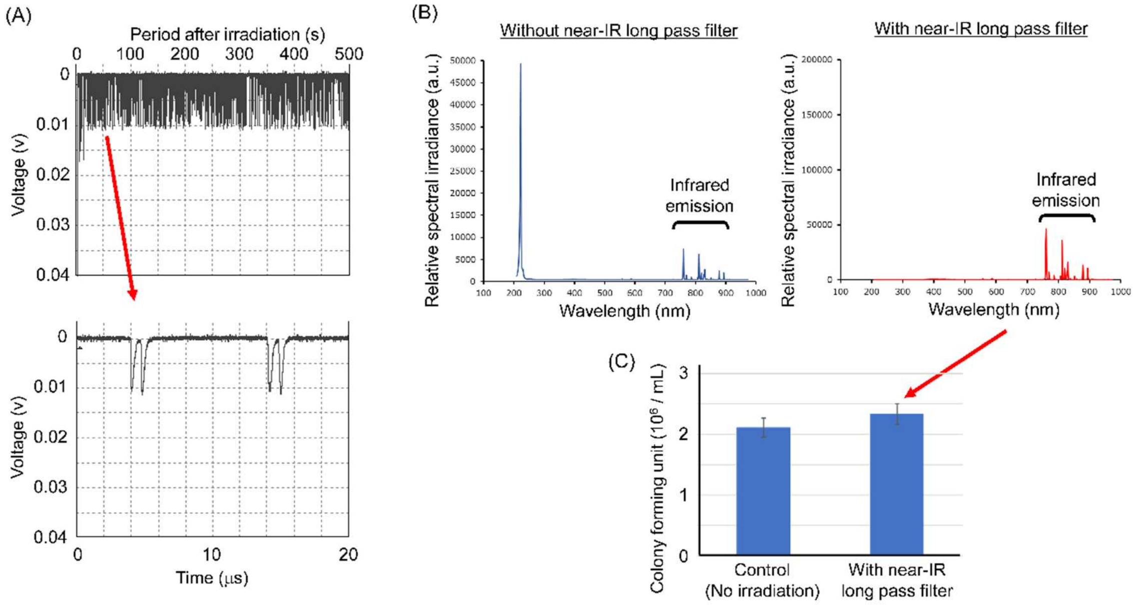

Supplementary Figure S3. Pulse emission and spectral irradiance of the filtered KrCl excimer lamp (222 nm). (A) Oscilloscope trace showing the pulsed emission of the filtered KrCl excimer lamp. The lower panel corresponds to the magnified waveform shown in the inset. (B) Relative spectral irradiance of the filtered KrCl excimer lamp with or without a near-infrared (IR) long-pass filter. (C) Evaluation of the antimicrobial effect of the filtered KrCl excimer lamp equipped with a short wavelength cutoff filter SCF-50S-37L (SIGMA KOKI, Tokyo, Japan). *Aspergillus fumigatus* MYA-4609 was irradiated with a dose of 10 mJ/cm^2 IR, and the antimicrobial effect was assessed by a colony-forming assay relative to the non-irradiated control.

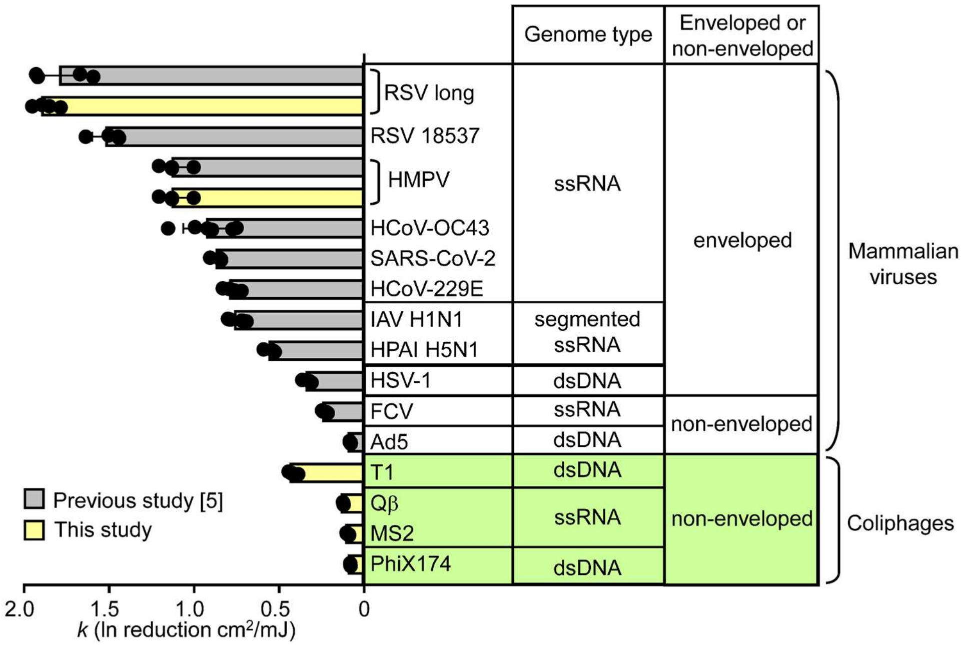

Supplementary Figure S4. Summary of viral genome or envelope type and inactivation rate constants (*k*) of mammalian viruses and coliphages irradiated with 281 nm light-emitting diodes (LEDs). The *k* was calculated as the natural logarithm of the reduction in plaque-forming units (PFU) divided by the irradiation fluence. The *k* values for mammalian viruses (gray bars) were referred from our previous study (Mawatari et al., 2025 [5]).
